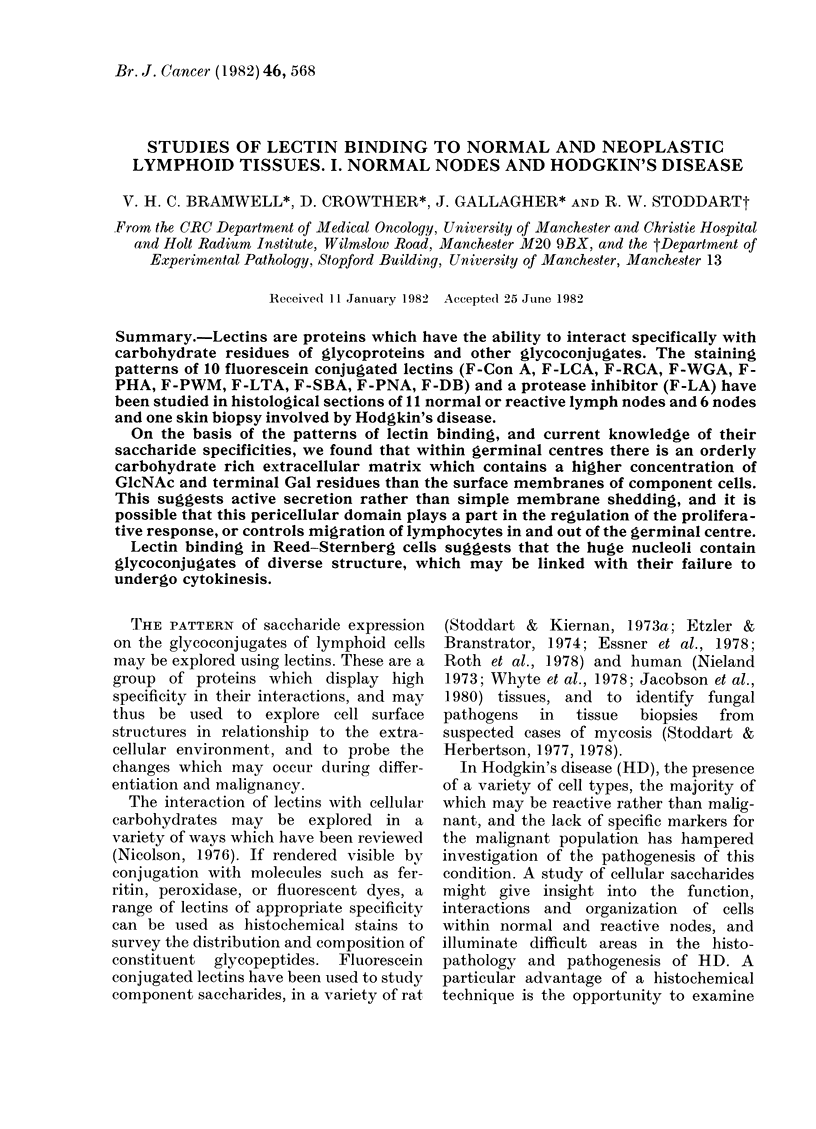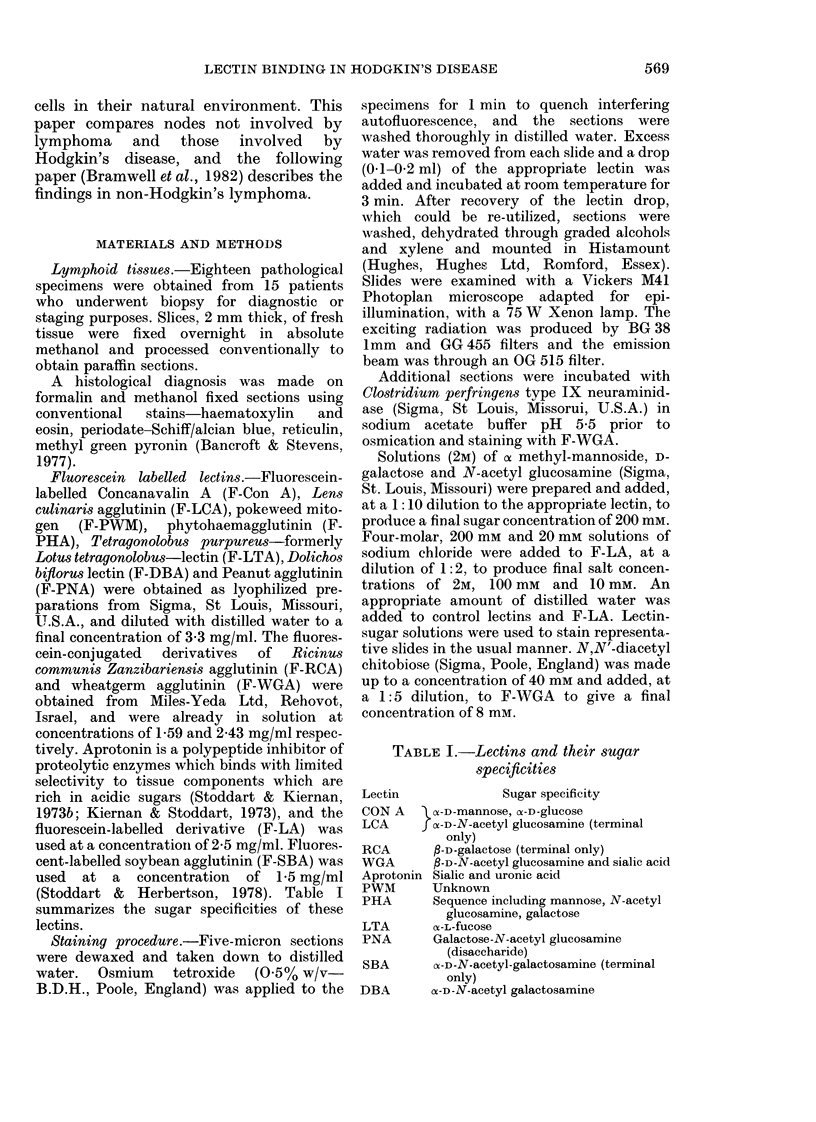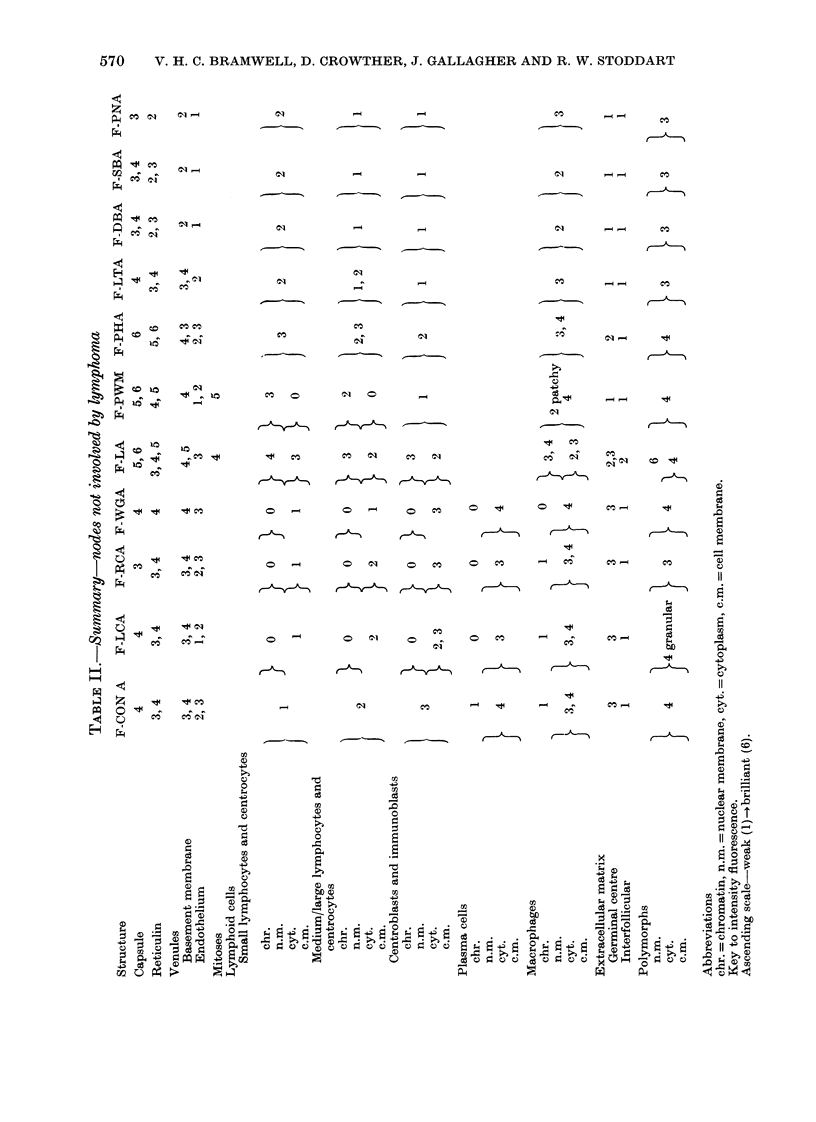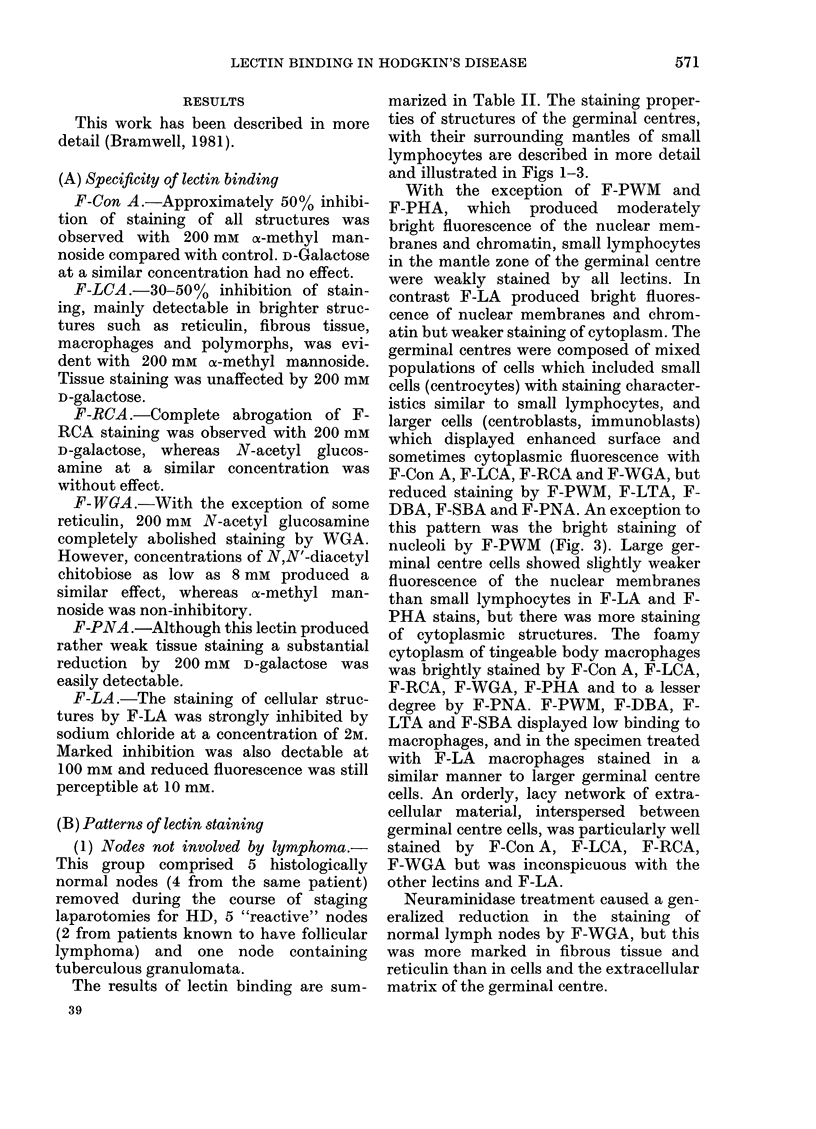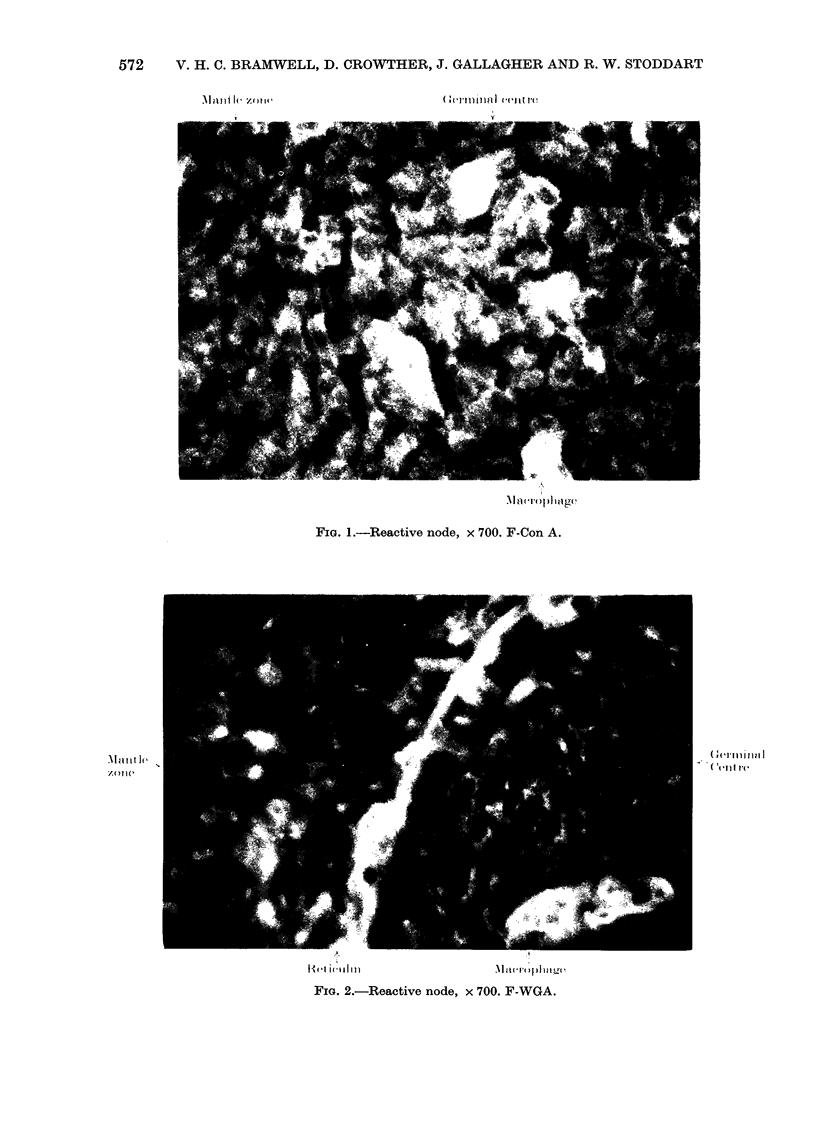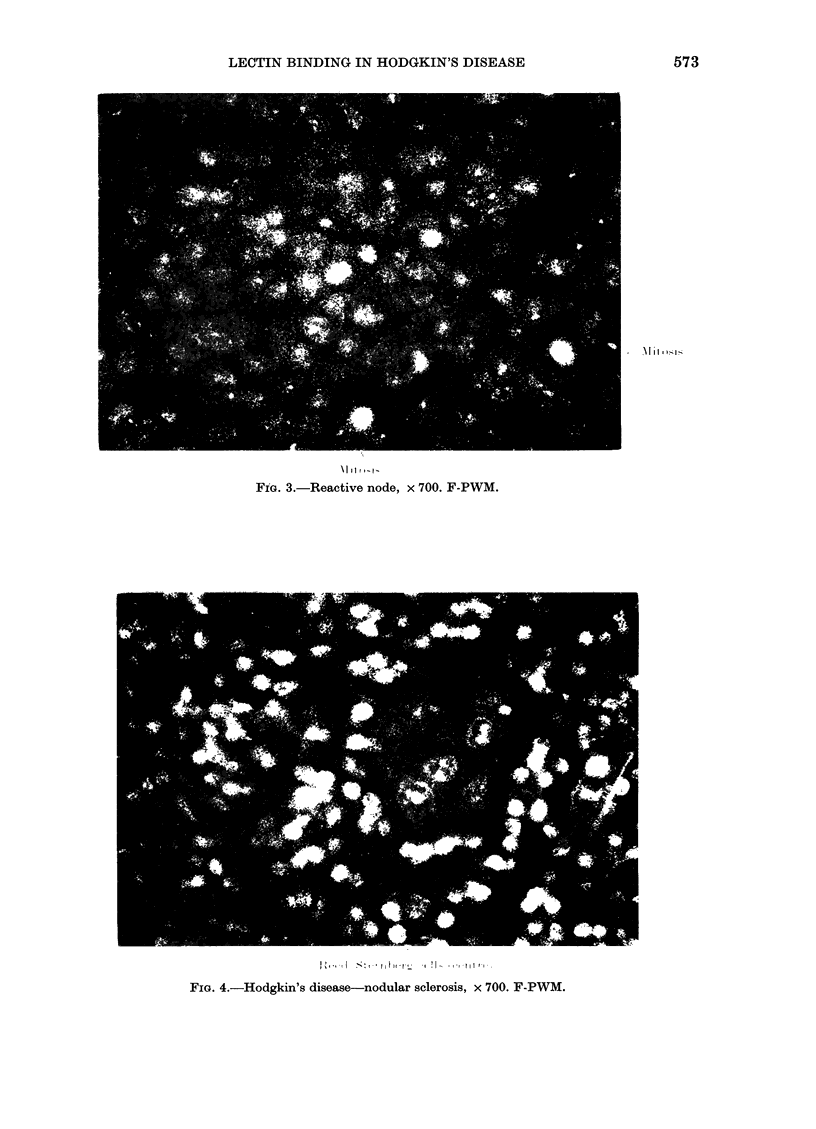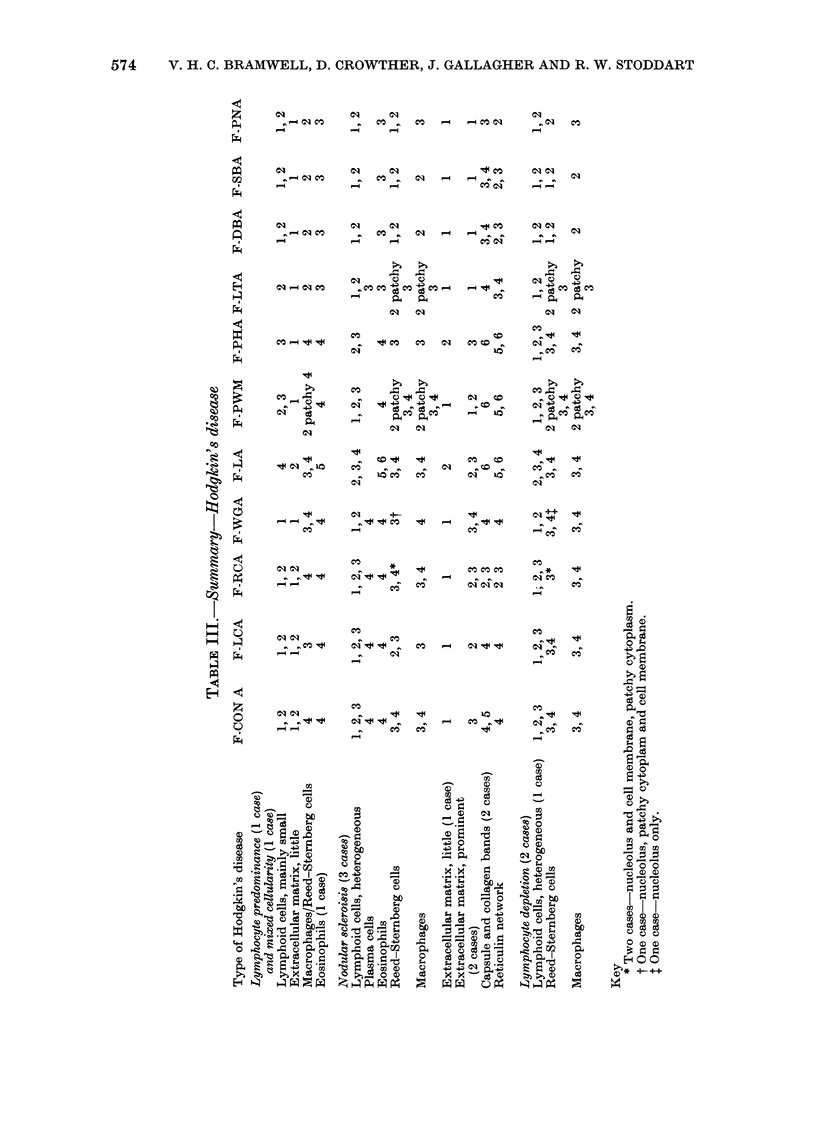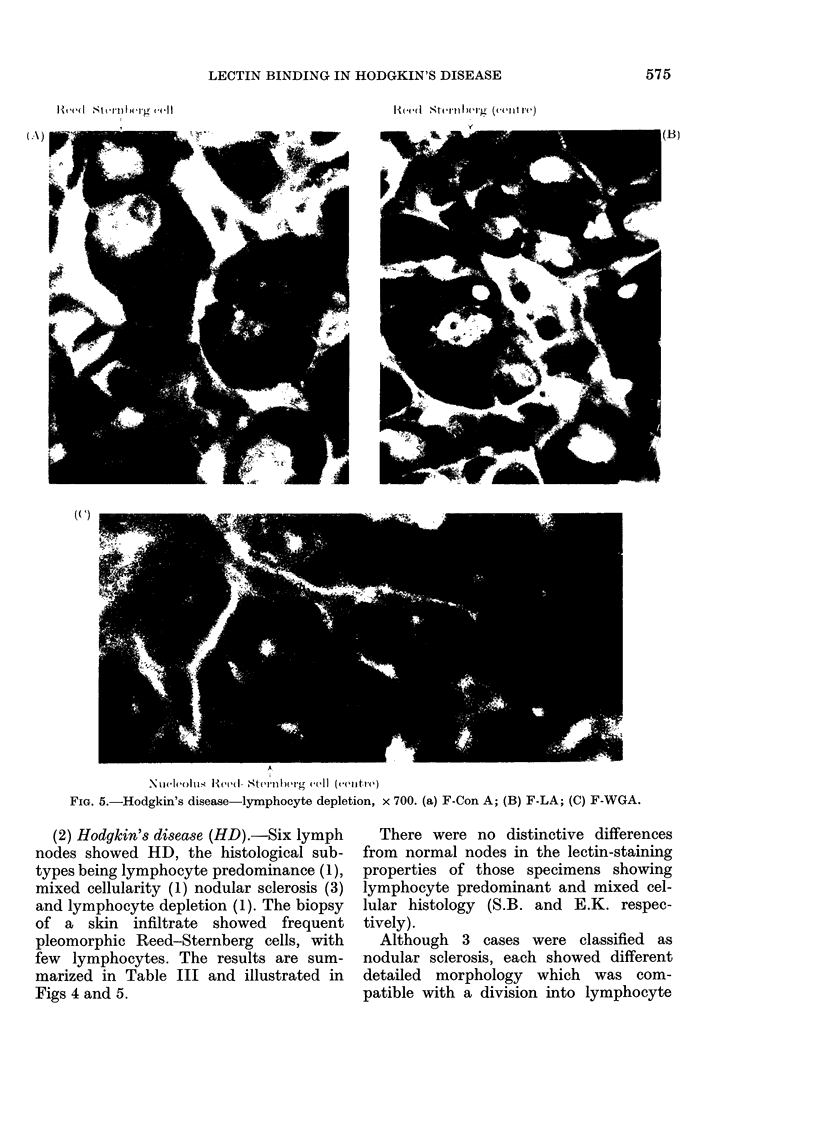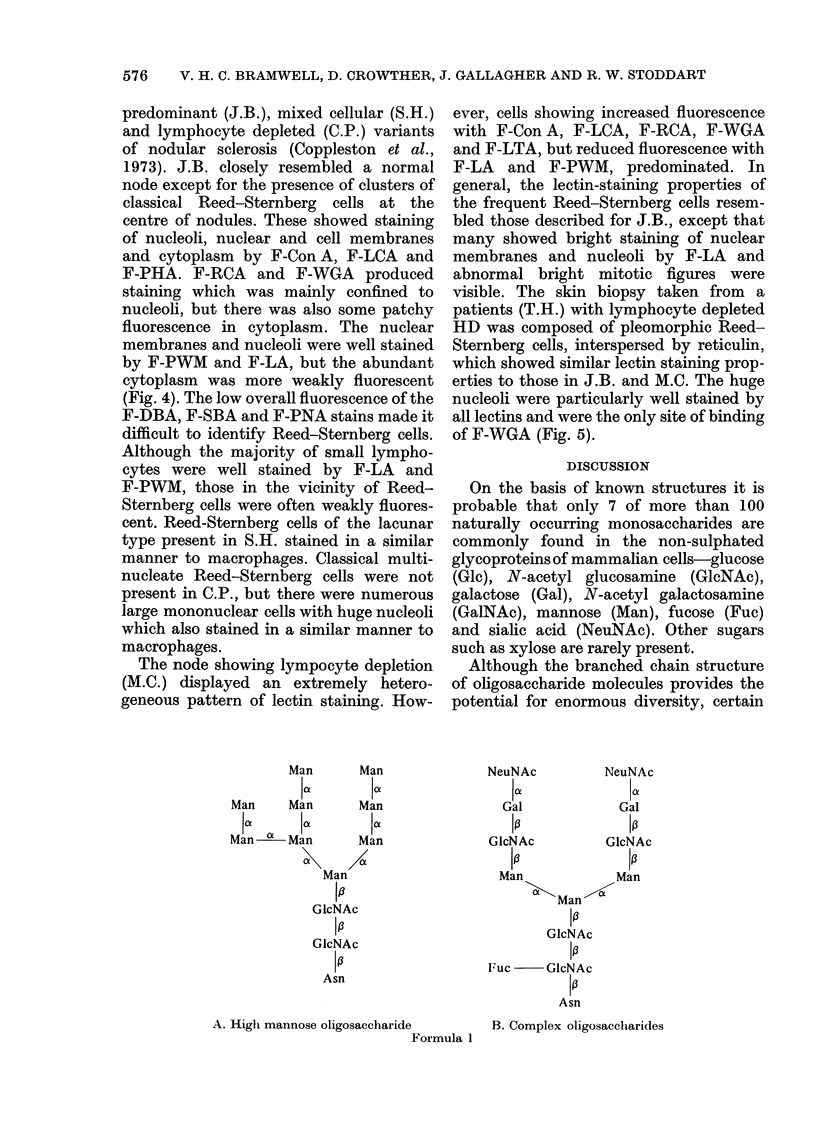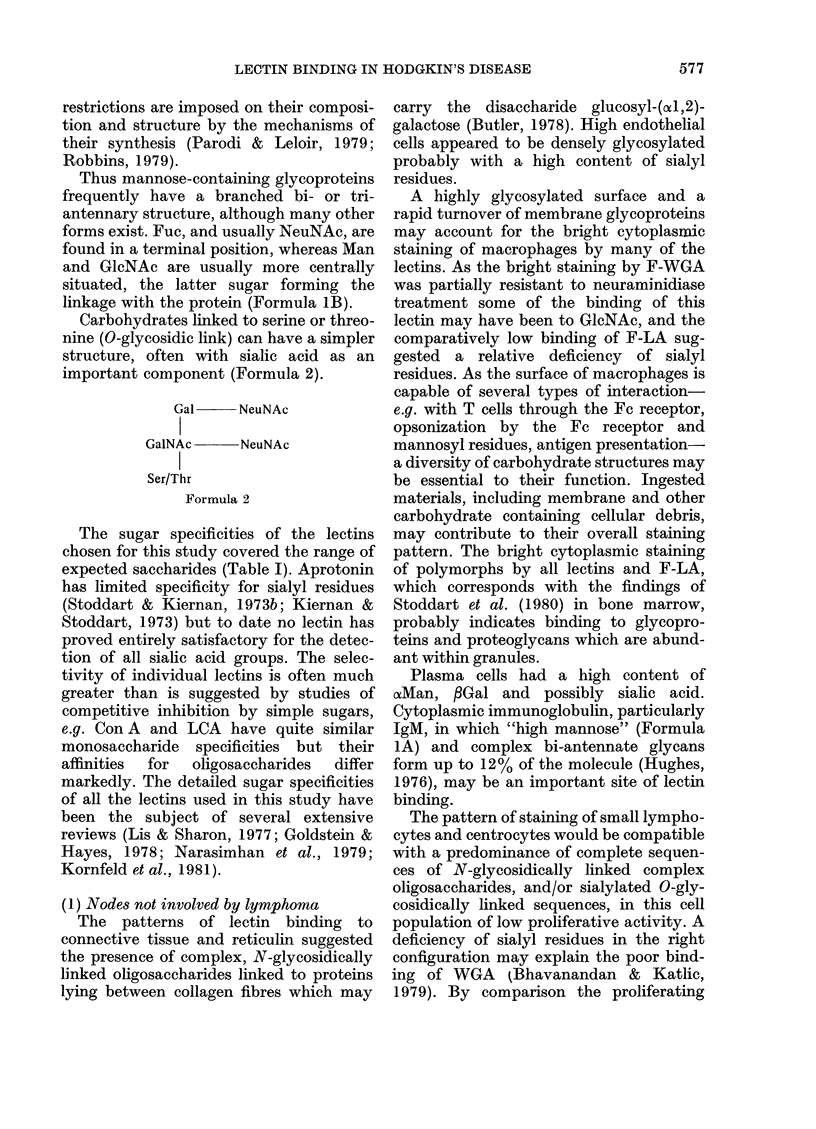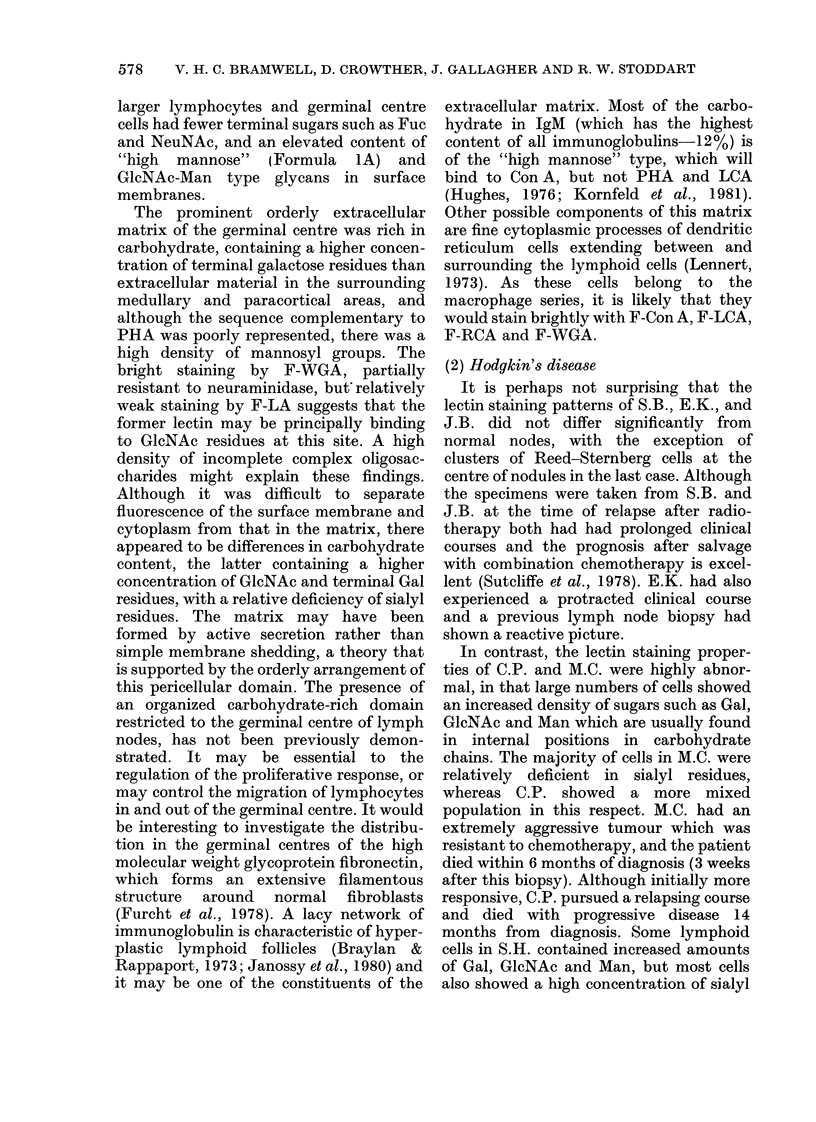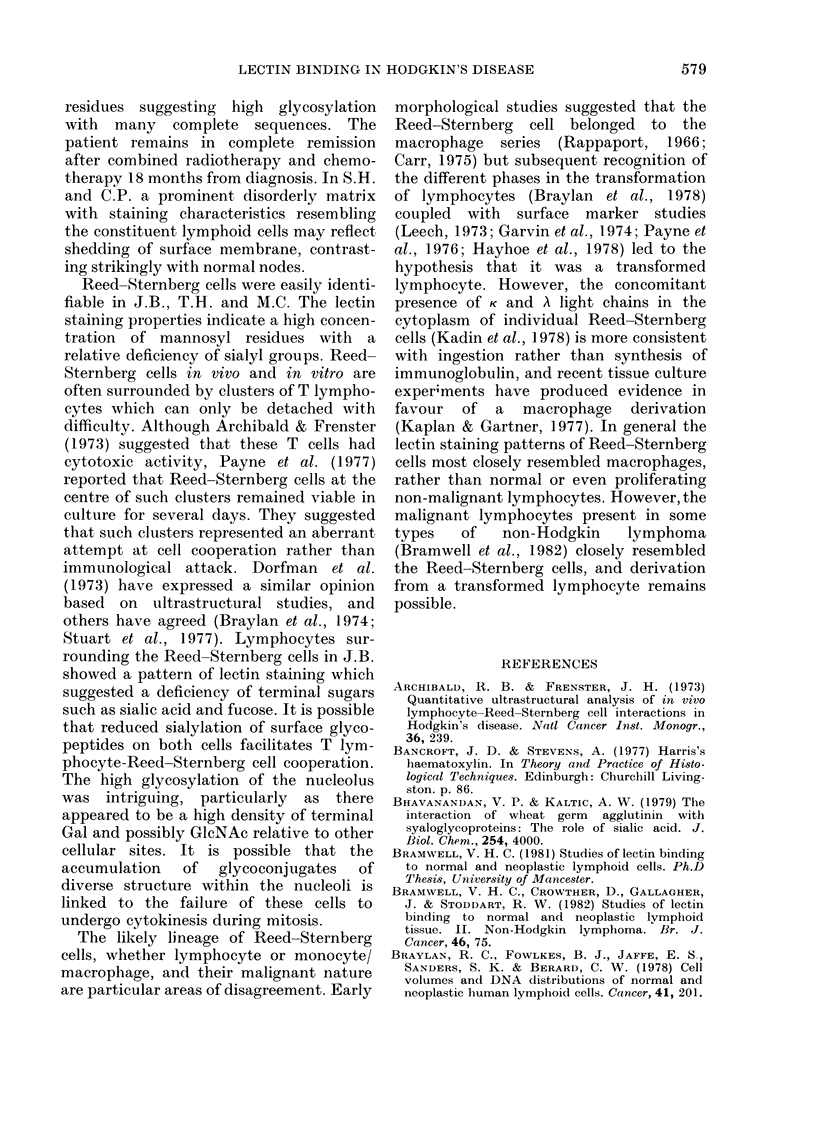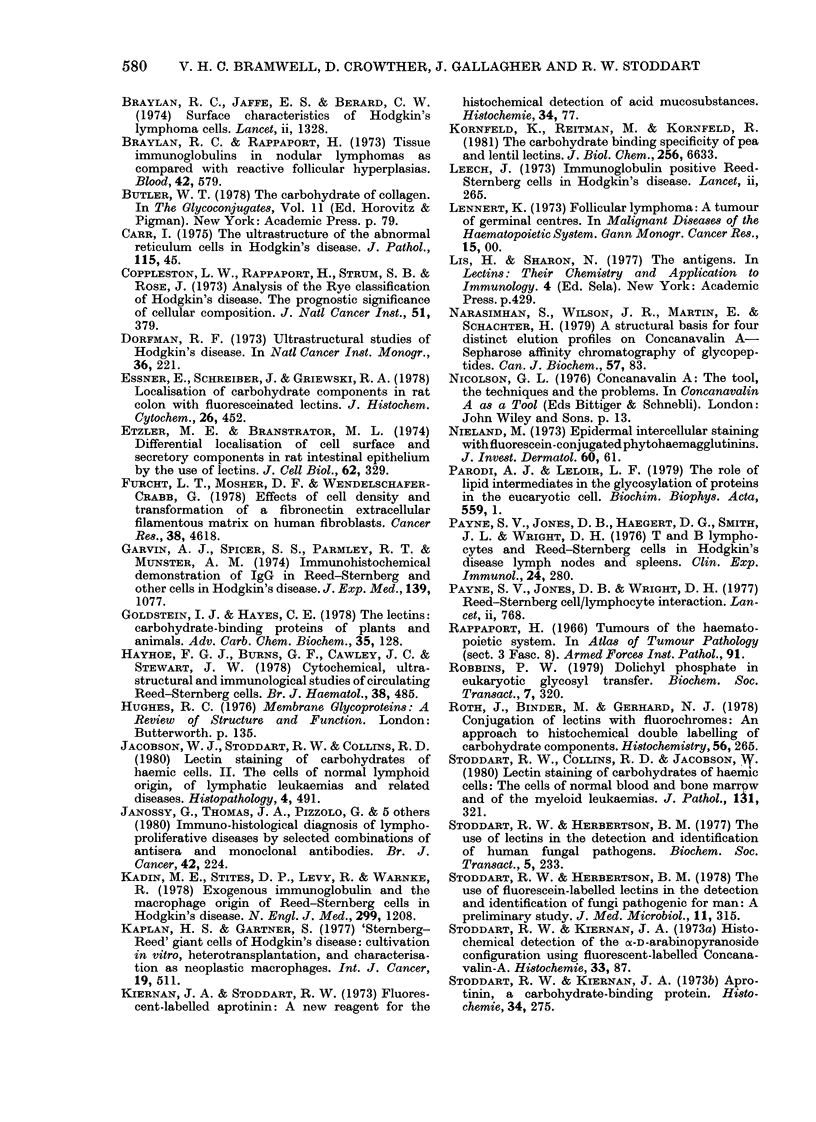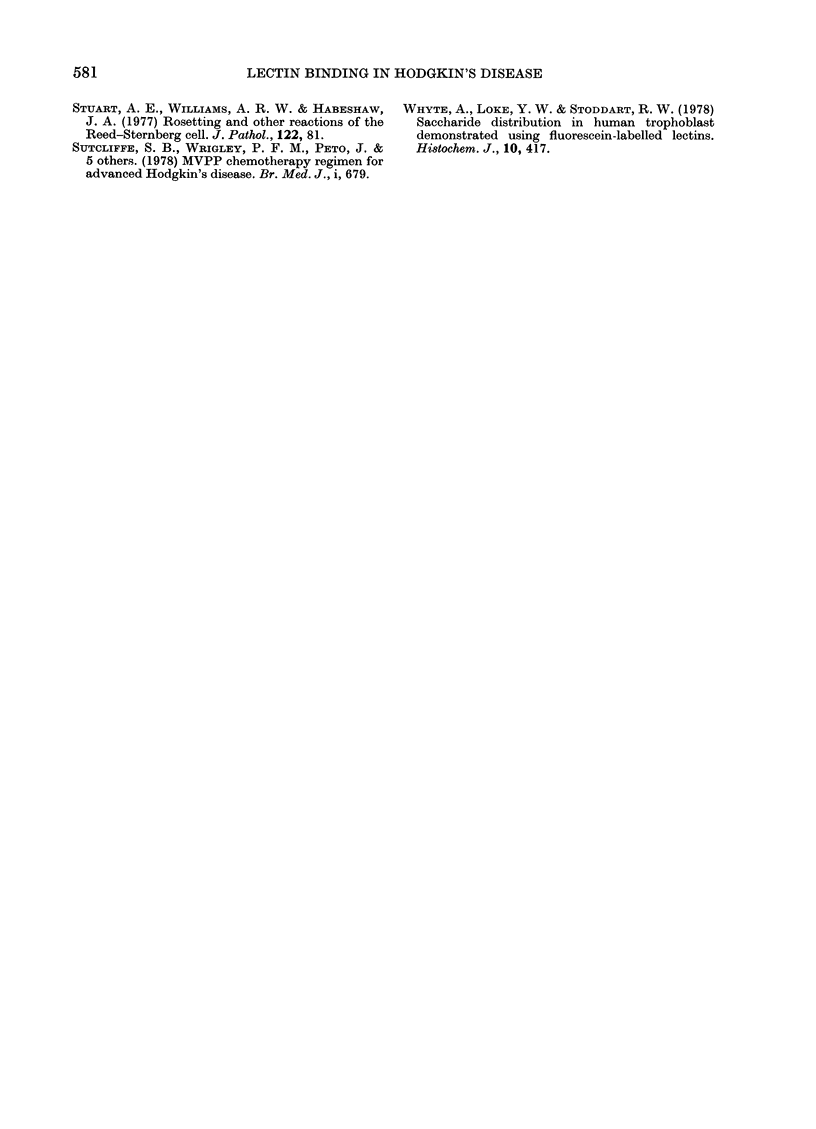# Studies of lectin binding to normal and neoplastic lymphoid tissues. I. Normal nodes and Hodgkin's disease.

**DOI:** 10.1038/bjc.1982.241

**Published:** 1982-10

**Authors:** V. H. Bramwell, D. Crowther, J. Gallagher, R. W. Stoddart

## Abstract

**Images:**


*Br. J. Cancer* (1982) **46**, 568

### STUDIES OF LECTIN BINDING TO NORMAL AND NEOPLASTIC LYMPHOID TISSUES. I. NORMAL NODES AND HODGKIN'S DISEASE

V. H. C. BRAMWELL*, D. CROWTHER*, J. GALLAGHER* AND R. W. STODDART†

*From the CRC Department of Medical Oncology, University of Manchester and Christie Hospital and Holt Radium Institute, Wilmslow Road, Manchester M20 9BX, and the †Department of Experimental Pathology, Stopford Building, University of Manchester, Manchester 13*

Received 11 January 1982 Accepted 25 June 1982

**Summary.—Lectins are proteins which have the ability to interact specifically with carbohydrate residues of glycoproteins and other glycoconjugates. The staining patterns of 10 fluorescein conjugated lectins (F-Con A, F-LCA, F-RCA, F-WGA, F-PHA, F-PWM, F-LTA, F-SBA, F-PNA, F-DB) and a protease inhibitor (F-LA) have been studied in histological sections of 11 normal or reactive lymph nodes and 6 nodes and one skin biopsy involved by Hodgkin's disease.**

**On the basis of the patterns of lectin binding, and current knowledge of their saccharide specificities, we found that within germinal centres there is an orderly carbohydrate rich extracellular matrix which contains a higher concentration of GlcNAc and terminal Gal residues than the surface membranes of component cells. This suggests active secretion rather than simple membrane shedding, and it is possible that this pericellular domain plays a part in the regulation of the proliferative response, or controls migration of lymphocytes in and out of the germinal centre.**

**Lectin binding in Reed–Sternberg cells suggests that the huge nucleoli contain glycoconjugates of diverse structure, which may be linked with their failure to undergo cytokinesis.**

THE PATTERN of saccharide expression on the glycoconjugates of lymphoid cells may be explored using lectins. These are a group of proteins which display high specificity in their interactions, and may thus be used to explore cell surface structures in relationship to the extracellular environment, and to probe the changes which may occur during differentiation and malignancy.

The interaction of lectins with cellular carbohydrates may be explored in a variety of ways which have been reviewed (Nicolson, 1976). If rendered visible by conjugation with molecules such as ferritin, peroxidase, or fluorescent dyes, a range of lectins of appropriate specificity can be used as histochemical stains to survey the distribution and composition of constituent glycopeptides. Fluorescein conjugated lectins have been used to study component saccharides, in a variety of rat (Stoddart & Kiernan, 1973*a*; Etzler & Branstrator, 1974; Essner *et al.*, 1978; Roth *et al.*, 1978) and human (Nieland 1973; Whyte *et al.*, 1978; Jacobson *et al.*, 1980) tissues, and to identify fungal pathogens in tissue biopsies from suspected cases of mycosis (Stoddart & Herbertson, 1977, 1978).

In Hodgkin's disease (HD), the presence of a variety of cell types, the majority of which may be reactive rather than malignant, and the lack of specific markers for the malignant population has hampered investigation of the pathogenesis of this condition. A study of cellular saccharides might give insight into the function, interactions and organization of cells within normal and reactive nodes, and illuminate difficult areas in the histopathology and pathogenesis of HD. A particular advantage of a histochemical technique is the opportunity to examine cells in their natural environment. This paper compares nodes not involved by lymphoma and those involved by Hodgkin's disease, and the following paper (Bramwell *et al.*, 1982) describes the findings in non-Hodgkin's lymphoma.

### MATERIALS AND METHODS

*Lymphoid tissues.*—Eighteen pathological specimens were obtained from 15 patients who underwent biopsy for diagnostic or staging purposes. Slices, 2 mm thick, of fresh tissue were fixed overnight in absolute methanol and processed conventionally to obtain paraffin sections.

A histological diagnosis was made on formalin and methanol fixed sections using conventional stains—haematoxylin and eosin, periodate–Schiff/alcian blue, reticulin, methyl green pyronin (Bancroft & Stevens, 1977).

*Fluorescein labelled lectins.*—Fluorescein-labelled Concanavalin A (F-Con A), *Lens culinaris* agglutinin (F-LCA), pokeweed mitogen (F-PWM), phytohaemagglutinin (F-PHA), *Tetragonolobus purpureus*—formerly *Lotus tetragonolobus*—lectin (F-LTA), *Dolichos biflorus* lectin (F-DBA) and Peanut agglutinin (F-PNA) were obtained as lyophilized preparations from Sigma, St Louis, Missouri, U.S.A., and diluted with distilled water to a final concentration of 3·3 mg/ml. The fluorescein-conjugated derivatives of *Ricinus communis Zanzibariensis* agglutinin (F-RCA) and wheatgerm agglutinin (F-WGA) were obtained from Miles-Yeda Ltd, Rehovot, Israel, and were already in solution at concentrations of 1·59 and 2·43 mg/ml respectively. Aprotonin is a polypeptide inhibitor of proteolytic enzymes which binds with limited selectivity to tissue components which are rich in acidic sugars (Stoddart & Kiernan, 1973*b*; Kiernan & Stoddart, 1973), and the fluorescein-labelled derivative (F-LA) was used at a concentration of 2·5 mg/ml. Fluorescent-labelled soybean agglutinin (F-SBA) was used at a concentration of 1·5 mg/ml (Stoddart & Herbertson, 1978). Table I summarizes the sugar specificities of these lectins.

*Staining procedure.*—Five-micron sections were dewaxed and taken down to distilled water. Osmium tetroxide (0·5% w/v—B.D.H., Poole, England) was applied to the specimens for 1 min to quench interfering autofluorescence, and the sections were washed thoroughly in distilled water. Excess water was removed from each slide and a drop (0·1–0·2 ml) of the appropriate lectin was added and incubated at room temperature for 3 min. After recovery of the lectin drop, which could be re-utilized, sections were washed, dehydrated through graded alcohols and xylene and mounted in Histamount (Hughes, Hughes Ltd, Romford, Essex). Slides were examined with a Vickers M41 Photoplan microscope adapted for epi-illumination, with a 75 W Xenon lamp. The exciting radiation was produced by BG 38 1mm and GG 455 filters and the emission beam was through an OG 515 filter.

Additional sections were incubated with *Clostridium perfringens* type IX neuraminidase (Sigma, St Louis, Missorui, U.S.A.) in sodium acetate buffer pH 5·5 prior to osmication and staining with F-WGA.

Solutions (2M) of α methyl-mannoside, D-galactose and *N*-acetyl glucosamine (Sigma, St. Louis, Missouri) were prepared and added, at a 1:10 dilution to the appropriate lectin, to produce a final sugar concentration of 200 mM. Four-molar, 200 mM and 20 mM solutions of sodium chloride were added to F-LA, at a dilution of 1:2, to produce final salt concentrations of 2M, 100 mM and 10 mM. An appropriate amount of distilled water was added to control lectins and F-LA. Lectin-sugar solutions were used to stain representative slides in the usual manner. *N,N'*-diacetyl chitobiose (Sigma, Poole, England) was made up to a concentration of 40 mM and added, at a 1:5 dilution, to F-WGA to give a final concentration of 8 mM.

TABLE I.—*Lectins and their sugar specificities*

| Lectin | Sugar specificity |
|---|---|
| CON A<br>LCA | α-D-mannose, α-D-glucose<br>α-D-*N*-acetyl glucosamine (terminal only) |
| RCA | β-D-galactose (terminal only) |
| WGA | β-D-*N*-acetyl glucosamine and sialic acid |
| Aprotonin | Sialic and uronic acid |
| PWM | Unknown |
| PHA | Sequence including mannose, *N*-acetyl glucosamine, galactose |
| LTA | α-L-fucose |
| PNA | Galactose-*N*-acetyl glucosamine (disaccharide) |
| SBA | α-D-*N*-acetyl-galactosamine (terminal only) |
| DBA | α-D-*N*-acetyl galactosamine |

TABLE II.—*Summary—nodes not involved by lymphoma*

| Structure | F-CON A | F-LCA | F-RCA | F-WGA | F-LA | F-PWM | F-PHA | F-LTA | F-DBA | F-SBA | F-PNA |
|---|---|---|---|---|---|---|---|---|---|---|---|
| Capsule | 4 | 4 | 3 | 4 | 5, 6 | 5, 6 | 6 | 4 | 3, 4 | 3, 4 | 3 |
| Reticulin | 3, 4 | 3, 4 | 3, 4 | 4 | 3, 4, 5 | 4, 5 | 5, 6 | 3, 4 | 2, 3 | 2, 3 | 2 |
| Venules | | | | | | | | | | | |
| Basement membrane | 3, 4 | 3, 4 | 3, 4 | 4 | 4, 5 | 4 | 4, 3 | 3, 4 | 2 | 2 | 2 |
| Endothelium | 2, 3 | 1, 2 | 2, 3 | 3 | 3 | 1, 2 | 2, 3 | 2 | 1 | 1 | 1 |
| Mitoses | | | | | 4 | 5 | | | | | |
| Lymphoid cells | | | | | | | | | | | |
| Small lymphocytes and centrocytes | | | | | | | | | | | |
| chr. | | 0 | 0 | 0 | 4 | 3 | | | | | |
| n.m. | 1 | | | | | | 3 | 2 | 2 | 2 | 2 |
| cyt. | | 1 | 1 | 1 | 3 | 0 | | | | | |
| c.m. | | | | | | | | | | | |
| Medium/large lymphocytes and centrocytes | | | | | | | | | | | |
| chr. | | 0 | 0 | 0 | 3 | 2 | | | | | |
| n.m. | 2 | | | | | | 2, 3 | 1, 2 | 1 | 1 | 1 |
| cyt. | | 2 | 2 | 1 | 2 | 0 | | | | | |
| c.m. | | | | | | | | | | | |
| Centroblasts and immunoblasts | | | | | | | | | | | |
| chr. | | 0 | 0 | 0 | 3 | | | | | | |
| n.m. | 3 | | | | | 1 | 2 | 1 | 1 | 1 | 1 |
| cyt. | | 2, 3 | 3 | 3 | 2 | | | | | | |
| c.m. | | | | | | | | | | | |
| Plasma cells | | | | | | | | | | | |
| chr. | 1 | 0 | 0 | 0 | | | | | | | |
| n.m. | | | | | | | | | | | |
| cyt. | 4 | 3 | 3 | 4 | | | | | | | |
| c.m. | | | | | | | | | | | |
| Macrophages | | | | | | | | | | | |
| chr. | 1 | 1 | 1 | 0 | 3, 4 | | | | | | |
| n.m. | | | | | | 2 patchy | 3, 4 | 3 | 2 | 2 | 3 |
| cyt. | 3, 4 | 3, 4 | 3, 4 | 4 | 2, 3 | 4 | | | | | |
| c.m. | | | | | | | | | | | |
| Extracellular matrix | | | | | | | | | | | |
| Germinal centre | 3 | 3 | 3 | 3 | 2,3 | 1 | 2 | 1 | 1 | 1 | 1 |
| Interfollicular | 1 | 1 | 1 | 1 | 2 | 1 | 1 | 1 | 1 | 1 | 1 |
| Polymorphs | | | | | | | | | | | |
| n.m. | | | | | 6 | | | | | | |
| cyt. | 4 | 4 granular | 3 | 4 | 4 | 4 | 4 | 3 | 3 | 3 | 3 |
| c.m. | | | | | | | | | | | |

Abbreviations chr. = chromatin, n.m. = nuclear membrane, cyt. = cytoplasm, c.m. = cell membrane.

Key to intensity fluorescence.

Ascending scale—weak (1)→brilliant (6).

RESULTS

This work has been described in more detail (Bramwell, 1981).

(A) *Specificity of lectin binding*

*F-Con A*.—Approximately 50% inhibition of staining of all structures was observed with 200 mM α-methyl mannoside compared with control. D-Galactose at a similar concentration had no effect.

*F-LCA*.—30–50% inhibition of staining, mainly detectable in brighter structures such as reticulin, fibrous tissue, macrophages and polymorphs, was evident with 200 mM α-methyl mannoside. Tissue staining was unaffected by 200 mM D-galactose.

*F-RCA*.—Complete abrogation of F-RCA staining was observed with 200 mM D-galactose, whereas *N*-acetyl glucosamine at a similar concentration was without effect.

*F-WGA*.—With the exception of some reticulin, 200 mM *N*-acetyl glucosamine completely abolished staining by WGA. However, concentrations of *N,N'*-diacetyl chitobiose as low as 8 mM produced a similar effect, whereas α-methyl mannoside was non-inhibitory.

*F-PNA*.—Although this lectin produced rather weak tissue staining a substantial reduction by 200 mM D-galactose was easily detectable.

*F-LA*.—The staining of cellular structures by F-LA was strongly inhibited by sodium chloride at a concentration of 2M. Marked inhibition was also dectable at 100 mM and reduced fluorescence was still perceptible at 10 mM.

(B) *Patterns of lectin staining*

(1) *Nodes not involved by lymphoma*.—This group comprised 5 histologically normal nodes (4 from the same patient) removed during the course of staging laparotomies for HD, 5 "reactive" nodes (2 from patients known to have follicular lymphoma) and one node containing tuberculous granulomata.

The results of lectin binding are summarized in Table II. The staining properties of structures of the germinal centres, with their surrounding mantles of small lymphocytes are described in more detail and illustrated in Figs 1–3.

With the exception of F-PWM and F-PHA, which produced moderately bright fluorescence of the nuclear membranes and chromatin, small lymphocytes in the mantle zone of the germinal centre were weakly stained by all lectins. In contrast F-LA produced bright fluorescence of nuclear membranes and chromatin but weaker staining of cytoplasm. The germinal centres were composed of mixed populations of cells which included small cells (centrocytes) with staining characteristics similar to small lymphocytes, and larger cells (centroblasts, immunoblasts) which displayed enhanced surface and sometimes cytoplasmic fluorescence with F-Con A, F-LCA, F-RCA and F-WGA, but reduced staining by F-PWM, F-LTA, F-DBA, F-SBA and F-PNA. An exception to this pattern was the bright staining of nucleoli by F-PWM (Fig. 3). Large germinal centre cells showed slightly weaker fluorescence of the nuclear membranes than small lymphocytes in F-LA and F-PHA stains, but there was more staining of cytoplasmic structures. The foamy cytoplasm of tingeable body macrophages was brightly stained by F-Con A, F-LCA, F-RCA, F-WGA, F-PHA and to a lesser degree by F-PNA. F-PWM, F-DBA, F-LTA and F-SBA displayed low binding to macrophages, and in the specimen treated with F-LA macrophages stained in a similar manner to larger germinal centre cells. An orderly, lacy network of extracellular material, interspersed between germinal centre cells, was particularly well stained by F-Con A, F-LCA, F-RCA, F-WGA but was inconspicuous with the other lectins and F-LA.

Neuraminidase treatment caused a generalized reduction in the staining of normal lymph nodes by F-WGA, but this was more marked in fibrous tissue and reticulin than in cells and the extracellular matrix of the germinal centre.

FIG. 1.—Reactive node, × 700. F-Con A.

FIG. 2.—Reactive node, × 700. F-WGA.

FIG. 3.—Reactive node, × 700. F-PWM.

FIG. 4.—Hodgkin's disease—nodular sclerosis, × 700. F-PWM.

TABLE III.—*Summary—Hodgkin's disease*

| Type of Hodgkin's disease | F-CON A | F-LCA | F-RCA | F-WGA | F-LA | F-PWM | F-PHA | F-LTA | F-DBA | F-SBA | F-PNA |
|---|---|---|---|---|---|---|---|---|---|---|---|
| *Lymphocyte predominance* (1 *case*) *and mixed cellularity* (1 *case*) | | | | | | | | | | | |
| Lymphoid cells, mainly small | 1, 2 | 1, 2 | 1, 2 | 1 | 4 | 2, 3 | 3 | 2 | 1, 2 | 1, 2 | 1, 2 |
| Extracellular matrix, little | 1, 2 | 1, 2 | 1, 2 | 1 | 2 | 1 | 1 | 1 | 1 | 1 | 1 |
| Macrophages/Reed–Sternberg cells | 4 | 3 | 4 | 3, 4 | 3, 4 | 2 patchy 4 | 4 | 2 | 2 | 2 | 2 |
| Eosinophils (1 case) | 4 | 4 | 4 | 4 | 5 | 4 | 4 | 3 | 3 | 3 | 3 |
| *Nodular scleroisis* (3 *cases*) | | | | | | | | | | | |
| Lymphoid cells, heterogeneous | 1, 2, 3 | 1, 2, 3 | 1, 2, 3 | 1, 2 | 2, 3, 4 | 1, 2, 3 | 2, 3 | 1, 2 | 1, 2 | 1, 2 | 1, 2 |
| Plasma cells | 4 | 4 | 4 | 4 | | | | 3 | | | |
| Eosinophils | 4 | 4 | 4 | 4 | 5, 6 | 4 | 4 | 3 | 3 | 3 | 3 |
| Reed–Sternberg cells | 3, 4 | 2, 3 | 3, 4* | 3† | 3, 4 | 2 patchy 3, 4 | 3 | 2 patchy 3 | 1, 2 | 1, 2 | 1, 2 |
| Macrophages | 3, 4 | 3 | 3, 4 | 4 | 3, 4 | 2 patchy 3, 4 | 3 | 2 patchy 3 | 2 | 2 | 3 |
| Extracellular matrix, little (1 case) | 1 | 1 | 1 | 1 | 2 | 1 | 2 | 1 | 1 | 1 | 1 |
| Extracellular matrix, prominent (2 cases) | 3 | 2 | 2, 3 | 3, 4 | 2, 3 | 1, 2 | 3 | 1 | 1 | 1 | 1 |
| Capsule and collagen bands (2 cases) | 4, 5 | 4 | 2, 3 | 4 | 6 | 6 | 6 | 4 | 3, 4 | 3, 4 | 3 |
| Reticulin network | 4 | 4 | 2 3 | 4 | 5, 6 | 5, 6 | 5, 6 | 3, 4 | 2, 3 | 2, 3 | 2 |
| *Lymphocyte depletion* (2 *cases*) | | | | | | | | | | | |
| Lymphoid cells, heterogeneous (1 case) | 1, 2, 3 | 1, 2, 3 | 1; 2, 3 | 1, 2 | 2, 3, 4 | 1, 2, 3 | 1, 2, 3 | 1, 2 | 1, 2 | 1, 2 | 1, 2 |
| Reed–Sternberg cells | 3, 4 | 3,4 | 3* | 3, 4‡ | 3, 4 | 2 patchy 3, 4 | 3, 4 | 2 patchy 3 | 1, 2 | 1, 2 | 2 |
| Macrophages | 3, 4 | 3, 4 | 3, 4 | 3, 4 | 3, 4 | 2 patchy 3, 4 | 3, 4 | 2 patchy 3 | 2 | 2 | 3 |

Key

* Two cases—nucleolus and cell membrane, patchy cytoplasm.

† One case—nucleolus, patchy cytoplam and cell membrane.

‡ One case—nucleolus only.

FIG. 5.—Hodgkin's disease—lymphocyte depletion, × 700. (a) F-Con A; (B) F-LA; (C) F-WGA.

(2) *Hodgkin's disease* (*HD*).—Six lymph nodes showed HD, the histological subtypes being lymphocyte predominance (1), mixed cellularity (1) nodular sclerosis (3) and lymphocyte depletion (1). The biopsy of a skin infiltrate showed frequent pleomorphic Reed–Sternberg cells, with few lymphocytes. The results are summarized in Table III and illustrated in Figs 4 and 5.

There were no distinctive differences from normal nodes in the lectin-staining properties of those specimens showing lymphocyte predominant and mixed cellular histology (S.B. and E.K. respectively).

Although 3 cases were classified as nodular sclerosis, each showed different detailed morphology which was compatible with a division into lymphocyte predominant (J.B.), mixed cellular (S.H.) and lymphocyte depleted (C.P.) variants of nodular sclerosis (Coppleston *et al.*, 1973). J.B. closely resembled a normal node except for the presence of clusters of classical Reed–Sternberg cells at the centre of nodules. These showed staining of nucleoli, nuclear and cell membranes and cytoplasm by F-Con A, F-LCA and F-PHA. F-RCA and F-WGA produced staining which was mainly confined to nucleoli, but there was also some patchy fluorescence in cytoplasm. The nuclear membranes and nucleoli were well stained by F-PWM and F-LA, but the abundant cytoplasm was more weakly fluorescent (Fig. 4). The low overall fluorescence of the F-DBA, F-SBA and F-PNA stains made it difficult to identify Reed–Sternberg cells. Although the majority of small lymphocytes were well stained by F-LA and F-PWM, those in the vicinity of Reed–Sternberg cells were often weakly fluorescent. Reed-Sternberg cells of the lacunar type present in S.H. stained in a similar manner to macrophages. Classical multinucleate Reed–Sternberg cells were not present in C.P., but there were numerous large mononuclear cells with huge nucleoli which also stained in a similar manner to macrophages.

The node showing lympocyte depletion (M.C.) displayed an extremely heterogeneous pattern of lectin staining. However, cells showing increased fluorescence with F-Con A, F-LCA, F-RCA, F-WGA and F-LTA, but reduced fluorescence with F-LA and F-PWM, predominated. In general, the lectin-staining properties of the frequent Reed–Sternberg cells resembled those described for J.B., except that many showed bright staining of nuclear membranes and nucleoli by F-LA and abnormal bright mitotic figures were visible. The skin biopsy taken from a patients (T.H.) with lymphocyte depleted HD was composed of pleomorphic Reed–Sternberg cells, interspersed by reticulin, which showed similar lectin staining properties to those in J.B. and M.C. The huge nucleoli were particularly well stained by all lectins and were the only site of binding of F-WGA (Fig. 5).

### DISCUSSION

On the basis of known structures it is probable that only 7 of more than 100 naturally occurring monosaccharides are commonly found in the non-sulphated glycoproteins of mammalian cells—glucose (Glc), *N*-acetyl glucosamine (GlcNAc), galactose (Gal), *N*-acetyl galactosamine (GalNAc), mannose (Man), fucose (Fuc) and sialic acid (NeuNAc). Other sugars such as xylose are rarely present.

Although the branched chain structure of oligosaccharide molecules provides the potential for enormous diversity, certain

A. High mannose oligosaccharide

B. Complex oligosaccharides

Formula 1

restrictions are imposed on their composition and structure by the mechanisms of their synthesis (Parodi & Leloir, 1979; Robbins, 1979).

Thus mannose-containing glycoproteins frequently have a branched bi- or triantennary structure, although many other forms exist. Fuc, and usually NeuNAc, are found in a terminal position, whereas Man and GlcNAc are usually more centrally situated, the latter sugar forming the linkage with the protein (Formula 1B).

Carbohydrates linked to serine or threonine (*O*-glycosidic link) can have a simpler structure, often with sialic acid as an important component (Formula 2).

Formula 2

The sugar specificities of the lectins chosen for this study covered the range of expected saccharides (Table I). Aprotonin has limited specificity for sialyl residues (Stoddart & Kiernan, 1973*b*; Kiernan & Stoddart, 1973) but to date no lectin has proved entirely satisfactory for the detection of all sialic acid groups. The selectivity of individual lectins is often much greater than is suggested by studies of competitive inhibition by simple sugars, *e.g.* Con A and LCA have quite similar monosaccharide specificities but their affinities for oligosaccharides differ markedly. The detailed sugar specificities of all the lectins used in this study have been the subject of several extensive reviews (Lis & Sharon, 1977; Goldstein & Hayes, 1978; Narasimhan *et al.*, 1979; Kornfeld *et al.*, 1981).

(1) *Nodes not involved by lymphoma*

The patterns of lectin binding to connective tissue and reticulin suggested the presence of complex, *N*-glycosidically linked oligosaccharides linked to proteins lying between collagen fibres which may carry the disaccharide glucosyl-($\alpha$1,2)-galactose (Butler, 1978). High endothelial cells appeared to be densely glycosylated probably with a high content of sialyl residues.

A highly glycosylated surface and a rapid turnover of membrane glycoproteins may account for the bright cytoplasmic staining of macrophages by many of the lectins. As the bright staining by F-WGA was partially resistant to neuraminidiase treatment some of the binding of this lectin may have been to GlcNAc, and the comparatively low binding of F-LA suggested a relative deficiency of sialyl residues. As the surface of macrophages is capable of several types of interaction—*e.g.* with T cells through the Fc receptor, opsonization by the Fc receptor and mannosyl residues, antigen presentation—a diversity of carbohydrate structures may be essential to their function. Ingested materials, including membrane and other carbohydrate containing cellular debris, may contribute to their overall staining pattern. The bright cytoplasmic staining of polymorphs by all lectins and F-LA, which corresponds with the findings of Stoddart *et al.* (1980) in bone marrow, probably indicates binding to glycoproteins and proteoglycans which are abundant within granules.

Plasma cells had a high content of $\alpha$Man, $\beta$Gal and possibly sialic acid. Cytoplasmic immunoglobulin, particularly IgM, in which "high mannose" (Formula 1A) and complex bi-antennate glycans form up to 12% of the molecule (Hughes, 1976), may be an important site of lectin binding.

The pattern of staining of small lymphocytes and centrocytes would be compatible with a predominance of complete sequences of *N*-glycosidically linked complex oligosaccharides, and/or sialylated *O*-glycosidically linked sequences, in this cell population of low proliferative activity. A deficiency of sialyl residues in the right configuration may explain the poor binding of WGA (Bhavanandan & Katlic, 1979). By comparison the proliferating larger lymphocytes and germinal centre cells had fewer terminal sugars such as Fuc and NeuNAc, and an elevated content of "high mannose" (Formula 1A) and GlcNAc-Man type glycans in surface membranes.

The prominent orderly extracellular matrix of the germinal centre was rich in carbohydrate, containing a higher concentration of terminal galactose residues than extracellular material in the surrounding medullary and paracortical areas, and although the sequence complementary to PHA was poorly represented, there was a high density of mannosyl groups. The bright staining by F-WGA, partially resistant to neuraminidase, but relatively weak staining by F-LA suggests that the former lectin may be principally binding to GlcNAc residues at this site. A high density of incomplete complex oligosaccharides might explain these findings. Although it was difficult to separate fluorescence of the surface membrane and cytoplasm from that in the matrix, there appeared to be differences in carbohydrate content, the latter containing a higher concentration of GlcNAc and terminal Gal residues, with a relative deficiency of sialyl residues. The matrix may have been formed by active secretion rather than simple membrane shedding, a theory that is supported by the orderly arrangement of this pericellular domain. The presence of an organized carbohydrate-rich domain restricted to the germinal centre of lymph nodes, has not been previously demonstrated. It may be essential to the regulation of the proliferative response, or may control the migration of lymphocytes in and out of the germinal centre. It would be interesting to investigate the distribution in the germinal centres of the high molecular weight glycoprotein fibronectin, which forms an extensive filamentous structure around normal fibroblasts (Furcht *et al.*, 1978). A lacy network of immunoglobulin is characteristic of hyperplastic lymphoid follicles (Braylan & Rappaport, 1973; Janossy *et al.*, 1980) and it may be one of the constituents of the extracellular matrix. Most of the carbohydrate in IgM (which has the highest content of all immunoglobulins—12%) is of the "high mannose" type, which will bind to Con A, but not PHA and LCA (Hughes, 1976; Kornfeld *et al.*, 1981). Other possible components of this matrix are fine cytoplasmic processes of dendritic reticulum cells extending between and surrounding the lymphoid cells (Lennert, 1973). As these cells belong to the macrophage series, it is likely that they would stain brightly with F-Con A, F-LCA, F-RCA and F-WGA.

(2) *Hodgkin's disease*

It is perhaps not surprising that the lectin staining patterns of S.B., E.K., and J.B. did not differ significantly from normal nodes, with the exception of clusters of Reed–Sternberg cells at the centre of nodules in the last case. Although the specimens were taken from S.B. and J.B. at the time of relapse after radiotherapy both had had prolonged clinical courses and the prognosis after salvage with combination chemotherapy is excellent (Sutcliffe *et al.*, 1978). E.K. had also experienced a protracted clinical course and a previous lymph node biopsy had shown a reactive picture.

In contrast, the lectin staining properties of C.P. and M.C. were highly abnormal, in that large numbers of cells showed an increased density of sugars such as Gal, GlcNAc and Man which are usually found in internal positions in carbohydrate chains. The majority of cells in M.C. were relatively deficient in sialyl residues, whereas C.P. showed a more mixed population in this respect. M.C. had an extremely aggressive tumour which was resistant to chemotherapy, and the patient died within 6 months of diagnosis (3 weeks after this biopsy). Although initially more responsive, C.P. pursued a relapsing course and died with progressive disease 14 months from diagnosis. Some lymphoid cells in S.H. contained increased amounts of Gal, GlcNAc and Man, but most cells also showed a high concentration of sialyl residues suggesting high glycosylation with many complete sequences. The patient remains in complete remission after combined radiotherapy and chemotherapy 18 months from diagnosis. In S.H. and C.P. a prominent disorderly matrix with staining characteristics resembling the constituent lymphoid cells may reflect shedding of surface membrane, contrasting strikingly with normal nodes.

Reed–Sternberg cells were easily identifiable in J.B., T.H. and M.C. The lectin staining properties indicate a high concentration of mannosyl residues with a relative deficiency of sialyl groups. Reed–Sternberg cells *in vivo* and *in vitro* are often surrounded by clusters of T lymphocytes which can only be detached with difficulty. Although Archibald & Frenster (1973) suggested that these T cells had cytotoxic activity, Payne *et al.* (1977) reported that Reed–Sternberg cells at the centre of such clusters remained viable in culture for several days. They suggested that such clusters represented an aberrant attempt at cell cooperation rather than immunological attack. Dorfman *et al.* (1973) have expressed a similar opinion based on ultrastructural studies, and others have agreed (Braylan *et al.*, 1974; Stuart *et al.*, 1977). Lymphocytes surrounding the Reed–Sternberg cells in J.B. showed a pattern of lectin staining which suggested a deficiency of terminal sugars such as sialic acid and fucose. It is possible that reduced sialylation of surface glycopeptides on both cells facilitates T lymphocyte-Reed–Sternberg cell cooperation. The high glycosylation of the nucleolus was intriguing, particularly as there appeared to be a high density of terminal Gal and possibly GlcNAc relative to other cellular sites. It is possible that the accumulation of glycoconjugates of diverse structure within the nucleoli is linked to the failure of these cells to undergo cytokinesis during mitosis.

The likely lineage of Reed–Sternberg cells, whether lymphocyte or monocyte/macrophage, and their malignant nature are particular areas of disagreement. Early morphological studies suggested that the Reed–Sternberg cell belonged to the macrophage series (Rappaport, 1966; Carr, 1975) but subsequent recognition of the different phases in the transformation of lymphocytes (Braylan *et al.*, 1978) coupled with surface marker studies (Leech, 1973; Garvin *et al.*, 1974; Payne *et al.*, 1976; Hayhoe *et al.*, 1978) led to the hypothesis that it was a transformed lymphocyte. However, the concomitant presence of $\kappa$ and $\lambda$ light chains in the cytoplasm of individual Reed–Sternberg cells (Kadin *et al.*, 1978) is more consistent with ingestion rather than synthesis of immunoglobulin, and recent tissue culture experiments have produced evidence in favour of a macrophage derivation (Kaplan & Gartner, 1977). In general the lectin staining patterns of Reed–Sternberg cells most closely resembled macrophages, rather than normal or even proliferating non-malignant lymphocytes. However, the malignant lymphocytes present in some types of non-Hodgkin lymphoma (Bramwell *et al.*, 1982) closely resembled the Reed–Sternberg cells, and derivation from a transformed lymphocyte remains possible.

### REFERENCES

Archibald, R. B. & Frenster, J. H. (1973) Quantitative ultrastructural analysis of *in vivo* lymphocyte–Reed–Sternberg cell interactions in Hodgkin's disease. *Natl Cancer Inst. Monogr.*, **36**, 239.

Bancroft, J. D. & Stevens, A. (1977) Harris's haematoxylin. In *Theory and Practice of Histological Techniques*. Edinburgh: Churchill Livingston. p. 86.

Bhavanandan, V. P. & Kaltic, A. W. (1979) The interaction of wheat germ agglutinin with syaloglycoproteins: The role of sialic acid. *J. Biol. Chem.*, **254**, 4000.

Bramwell, V. H. C. (1981) Studies of lectin binding to normal and neoplastic lymphoid cells. *Ph.D Thesis, University of Mancester.*

Bramwell, V. H. C., Crowther, D., Gallagher, J. & Stoddart, R. W. (1982) Studies of lectin binding to normal and neoplastic lymphoid tissue. II. Non-Hodgkin lymphoma. *Br. J. Cancer*, **46**, 75.

Braylan, R. C., Fowlkes, B. J., Jaffe, E. S., Sanders, S. K. & Berard, C. W. (1978) Cell volumes and DNA distributions of normal and neoplastic human lymphoid cells. *Cancer*, **41**, 201.

BRAYLAN, R. C., JAFFE, E. S. & BERARD, C. W. (1974) Surface characteristics of Hodgkin's lymphoma cells. *Lancet*, ii, 1328.

BRAYLAN, R. C. & RAPPAPORT, H. (1973) Tissue immunoglobulins in nodular lymphomas as compared with reactive follicular hyperplasias. *Blood*, **42**, 579.

BUTLER, W. T. (1978) The carbohydrate of collagen. In *The Glycoconjugates*, Vol. 11 (Ed. Horovitz & Pigman). New York: Academic Press. p. 79.

CARR, I. (1975) The ultrastructure of the abnormal reticulum cells in Hodgkin's disease. *J. Pathol.*, **115**, 45.

COPPLESTON, L. W., RAPPAPORT, H., STRUM, S. B. & ROSE, J. (1973) Analysis of the Rye classification of Hodgkin's disease. The prognostic significance of cellular composition. *J. Natl Cancer Inst.*, **51**, 379.

DORFMAN, R. F. (1973) Ultrastructural studies of Hodgkin's disease. In *Natl Cancer Inst. Monogr.*, **36**, 221.

ESSNER, E., SCHREIBER, J. & GRIEWSKI, R. A. (1978) Localisation of carbohydrate components in rat colon with fluoresceinated lectins. *J. Histochem. Cytochem.*, **26**, 452.

ETZLER, M. E. & BRANSTRATOR, M. L. (1974) Differential localisation of cell surface and secretory components in rat intestinal epithelium by the use of lectins. *J. Cell Biol.*, **62**, 329.

FURCHT, L. T., MOSHER, D. F. & WENDELSCHAFER-CRABB, G. (1978) Effects of cell density and transformation of a fibronectin extracellular filamentous matrix on human fibroblasts. *Cancer Res.*, **38**, 4618.

GARVIN, A. J., SPICER, S. S., PARMLEY, R. T. & MUNSTER, A. M. (1974) Immunohistochemical demonstration of IgG in Reed–Sternberg and other cells in Hodgkin's disease. *J. Exp. Med.*, **139**, 1077.

GOLDSTEIN, I. J. & HAYES, C. E. (1978) The lectins: carbohydrate-binding proteins of plants and animals. *Adv. Carb. Chem. Biochem.*, **35**, 128.

HAYHOE, F. G. J., BURNS, G. F., CAWLEY, J. C. & STEWART, J. W. (1978) Cytochemical, ultrastructural and immunological studies of circulating Reed–Sternberg cells. *Br. J. Haematol.*, **38**, 485.

HUGHES, R. C. (1976) *Membrane Glycoproteins: A Review of Structure and Function.* London: Butterworth. p. 135.

JACOBSON, W. J., STODDART, R. W. & COLLINS, R. D. (1980) Lectin staining of carbohydrates of haemic cells. II. The cells of normal lymphoid origin, of lymphatic leukaemias and related diseases. *Histopathology*, **4**, 491.

JANOSSY, G., THOMAS, J. A., PIZZOLO, G. & 5 others (1980) Immuno-histological diagnosis of lymphoproliferative diseases by selected combinations of antisera and monoclonal antibodies. *Br. J. Cancer*, **42**, 224.

KADIN, M. E., STITES, D. P., LEVY, R. & WARNKE, R. (1978) Exogenous immunoglobulin and the macrophage origin of Reed–Sternberg cells in Hodgkin's disease. *N. Engl. J. Med.*, **299**, 1208.

KAPLAN, H. S. & GARTNER, S. (1977) 'Sternberg–Reed' giant cells of Hodgkin's disease: cultivation *in vitro*, heterotransplantation, and characterisation as neoplastic macrophages. *Int. J. Cancer*, **19**, 511.

KIERNAN, J. A. & STODDART, R. W. (1973) Fluorescent-labelled aprotinin: A new reagent for the histochemical detection of acid mucosubstances. *Histochemie*, **34**, 77.

KORNFELD, K., REITMAN, M. & KORNFELD, R. (1981) The carbohydrate binding specificity of pea and lentil lectins. *J. Biol. Chem.*, **256**, 6633.

LEECH, J. (1973) Immunoglobulin positive Reed-Sternberg cells in Hodgkin's disease. *Lancet*, ii, 265.

LENNERT, K. (1973) Follicular lymphoma: A tumour of germinal centres. In *Malignant Diseases of the Haematopoietic System. Gann Monogr. Cancer Res.*, **15**, 00.

LIS, H. & SHARON, N. (1977) The antigens. In *Lectins: Their Chemistry and Application to Immunology.* **4** (Ed. Sela). New York: Academic Press. p.429.

NARASIMHAN, S., WILSON, J. R., MARTIN, E. & SCHACHTER, H. (1979) A structural basis for four distinct elution profiles on Concanavalin A—Sepharose affinity chromatography of glycopeptides. *Can. J. Biochem.*, **57**, 83.

NICOLSON, G. L. (1976) Concanavalin A: The tool, the techniques and the problems. In *Concanavalin A as a Tool* (Eds Bittiger & Schnebli). London: John Wiley and Sons. p. 13.

NIELAND, M. (1973) Epidermal intercellular staining with fluorescein-conjugated phytohaemagglutinins. *J. Invest. Dermatol.* **60**, 61.

PARODI, A. J. & LELOIR, L. F. (1979) The role of lipid intermediates in the glycosylation of proteins in the eucaryotic cell. *Biochim. Biophys. Acta*, **559**, 1.

PAYNE, S. V., JONES, D. B., HAEGERT, D. G., SMITH, J. L. & WRIGHT, D. H. (1976) T and B lymphocytes and Reed–Sternberg cells in Hodgkin's disease lymph nodes and spleens. *Clin. Exp. Immunol.*, **24**, 280.

PAYNE, S. V., JONES, D. B. & WRIGHT, D. H. (1977) Reed–Sternberg cell/lymphocyte interaction. *Lancet*, ii, 768.

RAPPAPORT, H. (1966) Tumours of the haematopoietic system. In *Atlas of Tumour Pathology* (sect. 3 Fasc. 8). *Armed Forces Inst. Pathol.*, **91**.

ROBBINS, P. W. (1979) Dolichyl phosphate in eukaryotic glycosyl transfer. *Biochem. Soc. Transact.*, **7**, 320.

ROTH, J., BINDER, M. & GERHARD, N. J. (1978) Conjugation of lectins with fluorochromes: An approach to histochemical double labelling of carbohydrate components. *Histochemistry*, **56**, 265.

STODDART, R. W., COLLINS, R. D. & JACOBSON, W. (1980) Lectin staining of carbohydrates of haemic cells: The cells of normal blood and bone marrow and of the myeloid leukaemias. *J. Pathol.*, **131**, 321.

STODDART, R. W. & HERBERTSON, B. M. (1977) The use of lectins in the detection and identification of human fungal pathogens. *Biochem. Soc. Transact.*, **5**, 233.

STODDART, R. W. & HERBERTSON, B. M. (1978) The use of fluorescein-labelled lectins in the detection and identification of fungi pathogenic for man: A preliminary study. *J. Med. Microbiol.*, **11**, 315.

STODDART, R. W. & KIERNAN, J. A. (1973*a*) Histochemical detection of the α-D-arabinopyranoside configuration using fluorescent-labelled Concanavalin-A. *Histochemie*, **33**, 87.

STODDART, R. W. & KIERNAN, J. A. (1973*b*) Aprotinin, a carbohydrate-binding protein. *Histochemie*, **34**, 275.

Stuart, A. E., Williams, A. R. W. & Habeshaw, J. A. (1977) Rosetting and other reactions of the Reed–Sternberg cell. *J. Pathol.*, **122,** 81.

Sutcliffe, S. B., Wrigley, P. F. M., Peto, J. & 5 others. (1978) MVPP chemotherapy regimen for advanced Hodgkin's disease. *Br. Med. J.*, i, 679.

Whyte, A., Loke, Y. W. & Stoddart, R. W. (1978) Saccharide distribution in human trophoblast demonstrated using fluorescein-labelled lectins. *Histochem. J.*, **10,** 417.